# *Lactiplantibacillus plantarum* Lp20 Alleviates High Fat Diet-Induced Obesity in Mice via Its Bile Salt Hydrolase Activity

**DOI:** 10.3390/nu17223555

**Published:** 2025-11-14

**Authors:** Xiaoyue Bai, Fangzhou Lu, Yizhi Jing, Hui Wang, Haidong Qian, Ming Zhang, Zhengyuan Zhai, Yanling Hao

**Affiliations:** 1 College of Food Science and Nutritional Engineering, China Agricultural University, Beijing 100083, China; baixiaoyue21@163.com (X.B.); lufangzhou@smart.org.cn (F.L.); wanghui06@sanyuan.com.cn (H.W.); 2 Key Laboratory of Precision Nutrition and Food Quality, Department of Nutrition and Health, China Agricultural University, Beijing 100190, China; j15502414985@163.com (Y.J.); qianhaidong2023@163.com (H.Q.); 3 School of Food and Health, Beijing Technology and Business University, Beijing 100048, China; zhangming@th.btbu.edu.cn

**Keywords:** *Lactiplantibacillus plantarum*, bile salt hydrolase, gene knock-out, anti-obesity

## Abstract

**Background:** Obesity is a highly prevalent chronic disease characterized by excessive weight gain and fat accumulation. There is growing evidence that *Lactiplantibacillus plantarum* strains with bile salt hydrolase (BSH) activity are effective in preventing and alleviating obesity. **Methods:** Initially, we screened bacterial strains with high hydrolytic activity against glycochenodeoxycholic acid (GDCA), and constructed an isogenic bsh1 knockout mutant. Subsequently, male C57BL/6J mice fed a high-fat diet (HFD) were randomly assigned to receive daily gavage of either the wild-type Lp20 (Lp20-WT) or the bsh1-deficient mutant (Lp20-*Δbsh1*) for 8 weeks. Serum cholesterol levels and histopathological changes in liver sections were monitored. Hepatic gene expression was quantified by RT-qPCR, and fecal bacterial communities were analyzed via 16S rRNA gene sequencing. These comprehensive assessments aimed to evaluate metabolic improvements and uncover the potential mechanisms behind the observed effects. **Results:** *L. plantarum* Lp20 hydrolyzed 91.62% of GDCA, exhibiting the highest bile-salt hydrolase (BSH) activity among tested isolates. Whole-genome sequencing and in-silico analyses mapped this activity to *bsh1*; gene deletion of *bsh1* confirmed the role of *bsh1* in GDCA hydrolysis. Daily gavage of the wild-type strain (Lp20-WT) to diet-induced obese mice markedly attenuated weight gain, reduced inguinal white adipose tissue and mesenteric fat mass, and lowered serum TC and LDL-C by 20.8% and 33.3%, respectively, while decreasing ALT and AST levels and reversing hepatic steatosis. In contrast, the bsh1-null mutant (Lp20-*Δbsh1*) failed to elicit any measurable metabolic benefit. Mechanistically, Lp20-WT upregulated rate-limiting bile-acid synthetic enzymes CYP7A1 and CYP27A1, thereby accelerating the catabolism of cholesterol into bile acids. Concurrently, it activated hepatic TGR5 and FXR signaling axes to modulate hepatic metabolism. Moreover, Lp20-WT restructured the gut microbiota by notably enhancing the abundance of beneficial bacteria such as *norank_f__Muribaculaceae*, *Akkermansia*, and *Alistipes*, while reducing the abundance of potentially harmful taxa, including *norank_f__Desulfovibrionaceae*, *Dubosiella*, and *Mucispirillum*. **Conclusions:** This study provides direct evidence of BSH’s anti-obesity effects through gene deletion. Specifically, BSH lowers cholesterol by modulating hepatic bile-acid metabolism-related gene expression and altering the gut microbiota composition. However, the study is limited by a small sample size (*n* = 6), the use of male mice only, and its preclinical stage, indicating a need for further validation across diverse strains and human populations.

## 1. Introduction

Obesity increased incidences of related comorbidities, such as type 2 diabetes, cardiovascular diseases and cancer [1,2]. According to the World Obesity Atlas 2023 report, the number of adults with obesity is projected to rise from 0.81 billion in 2020 to 1.53 billion in 2035. Most of these individuals will primarily reside in middle-income countries, which often have limited resources and infrastructure to address obesity adequately [3]. Low-harm, safe and effective intervention methods are urgently needed. Clinical studies have demonstrated that gut microbiota dysbiosis, which can trigger inflammation, is closely associated with obesity and its related health issues [4]. When fecal microbiota from obese individuals were transplanted into germ-free mice, the mice developed an obese phenotype, highlighting the critical role of microbiota disruption in obesity development [5]. In a cohort study, the supplementation with a probiotic containing two strains of *Bifidobacterium* and three strains of *Lactobacillus* (at a dosage of 500 billion CFU/day) for six months led to significant reductions in body weight, serum total cholesterol (TC), and low-density lipoprotein cholesterol (LDL-C) [6]. In another study, male Sprague-Dawley rats fed heat-inactivated or live *Lactobacillus reuteri* GMNL263 while on a high-fat diet (HFD) for 12 weeks showed marked decreases in body weight, serum glucose, and insulin levels [7]. Similarly, a 12-week intervention with *Lactiplantibacillus plantarum* Y44 not only alleviated lipid metabolism disorder by decreasing TC and LDL-C levels, but also reduced serum aspartate aminotransferase (AST) and alanine aminotransferase (ALT) in obese mice [8]. Probiotic intervention has emerged as a promising strategy for alleviating obesity. However, its efficacy is largely dependent on the bacterial strains employed. Thus, future probiotic trials should prioritize the selection and optimal pairing of strains to enhance intervention outcomes.

In recent years, there has been growing interest in the potential of modulating bile acid metabolism as a therapeutic approach for obesity and related metabolic disorders. Probiotics, as key microbial modulators of host metabolism, have been shown to play a role through specific metabolites or enzymes, such as bile salt hydrolase (BSH) [9,10]. Recent studies have highlighted the potential role of BSH in regulating lipid metabolism and ameliorating obesity-related syndrome. An in vitro experiment revealed that *L. plantarum* TCI378 with bile salts deconjugation ability reduced oil droplet accumulation and lipid metabolism in 3T3-L1 [11]. For in vivo assay, *L. plantarum* H-87 with a distinguished BSH activity exhibited a significant inhibitory effect on weight gain and lipid accumulation in HFD-fed mice [12]. Furthermore, the recombinant *Escherichia coli* MG1655 with *bsh*1 from *Lactobacillus salivarius* JCM1046 significantly reduced weight gain, LDL-C and triglyceride (TG) levels in obese mice [13]. *L. acidophilus* GOLDGUT-LA100 with high BSH activity was also demonstrate concrete anti-obesity benefit in HFD-induced obese mice [14].

Bile acids (BAs), synthesized in the liver, are stored within the gallbladder and released into the gastrointestinal tract following food ingestion [15]. BSH deconjugated tauro-conjugated or glycol-conjugated BAs in the small intestine, generating their corresponding free BAs [16]. The reduced solubility of these free BAs increases bile excretion, promoting the conversion of cholesterol to BAs. CYP7A1, the rate-limiting enzyme in bile acid biosynthesis, promotes hepatic cholesterol catabolism when expressed in the liver [17]. This effect contributes to the reduction of cholesterol levels and lipid absorption in the small intestine [18,19,20]. Due to this gateway efficacy, primary BAs can be further transformed into secondary bile acids (sBAs) by cecal and colonic microbiota [21,22]. Certain sBAs serve as ligands for multiple BA receptors, including the farnesoid X receptor (FXR) and Takeda G protein-coupled receptor (TGR5) [23]. Activation of FXR by deoxycholic acid (DCA) inhibits SREBP1c production, thereby reducing triglyceride synthesis and ameliorating hepatic steatosis [24,25,26]. As potent TGR5 agonists, lithocholic acid (LCA) and DCA stimulate intestinal GLP-1 secretion, thereby enhancing insulin sensitivity and glucose homeostasis in obese individuals [24]. Probiotic strains with high BSH activity have the potential to modulate bile acid composition, thereby influencing the activity of signaling pathways such as FXR and TGR5 and contributing to metabolic regulation.

In this study, Lp20, exhibiting the highest BSH activity among 23 BSH-positive strains, was selected and confirmed to be safe through hemolytic and antibiotic resistance tests. Genomic sequencing and bioinformatics analysis indicated that gene *bsh1* was associated with BSH activity of Lp20. Subsequently, a *bsh1* mutant strain of Lp20 was constructed by homologous double-overcrossing recombination. The Lp20-Δ*bsh1* strain was used as the negative control for Lp20 to investigate the correlation between probiotic BSH activity and its efficacy in reducing fat in the high fat diet-induced obese mice.

## 2. Materials and Methods

### 2.1. Bacterial Strains and Culture Condition

The bacterial strains and plasmids used in this study are listed in Table S1. The *L. plantarum* strain was anaerobically cultured at 37 °C in de Man, Rogosa, and Sharpe (MRS) medium. *Escherichia coli* DH5α, was grown at 37 °C in Luria–Bertani (LB) broth for 12 h with shaking at 220 rpm. The *Lactococcus lactis* NZ9000 strain was cultivated at 30 °C in M17 medium (Oxoid, Unipath, Basingstoke, UK) supplemented with 0.5% wt/vol glucose (GM17) for 14 h. When required, the media were supplemented with the appropriate antibiotics at the following concentrations: 100 µg/mL ampicillin for *E. coli*, 10 µg/mL chloramphenicol for *L. lactis* and 10 µg/mL erythromycin for *L. plantarum*.

### 2.2. BSH Activity Assays of L. plantarum

For the qualitative assay of BSH activity, the bile salt plate method was employed in this study [27,28]. Briefly, MRS agar plate was supplemented with 0.1% wt/vol glycodeoxycholic acid (GDCA, Sigma Aldrich, Saint Louis, MO, USA) and 0.1% wt/vol taurodeoxycholic acid (TDCA, Sigma Aldrich), respectively. The 23 *L. plantarum* strains were streaked onto the plates and subjected to anaerobic culture for 48 h, with three technical replicates. Positive results were indicated by the formation of a halo-like precipitate surrounding the colonies.

BSH activity was also analyzed quantitatively by the ninhydrin assay, which was a colorimetric test to determine the amount of amino acids released from individual BAs through BSH deconjugation [29,30,31]. Bacterial cells in the stationary phase were collected by centrifugation, washed with sodium phosphate buffer, and resuspended to OD_600nm_ = 5.0. A 50 μL cell suspension was mixed with an equal volume of 20 mM bile salt substrate and incubated at 37 °C for 1 h. The reaction was terminated by the addition of 100 μL trichloroacetic acid. Then the mixture was centrifuged to collect 100 μL supernatant, which was then mixed with 900 μL ninhydrin. The reaction system was carried out in boiling water bath for 14 min and immediately transferred to an ice bath for color stabilization. Based on a standard curve, the OD at 570 nm was measured to determine the amino acid concentration. A standard curve was generated by plotting glycine concentration (mM) on the x-axis and OD570 values on the y-axis, and a regression equation was calculated. The concentration of amino acids generated from the hydrolysis of 5 mM conjugated bile salt substrate was determined using this standard curve, and the amount of bile salt hydrolysis was then calculated. BSH activity assays for each *L. plantarum* strain were performed in triplicate.

The bile salt hydrolysis rate was calculated using the following formula:

$$Bile\;salt\;hydrolysis\;rate\;(\%)\;=\;\frac{C}{5}\times 100\%$$

5 stands for the initial concentration of bile salt substrates in the reaction system is 5 mM, *C* is the concentration of amino acids after substrate hydrolysis.

### 2.3. Draft Genome Sequencing and Comparative Analysis

The genome of Lp20 was extracted using the Bacterial Genome Extraction Kit (Tiangen, Beijing, China). Purified genome was sequenced and assembled by Beijing Novogene Bioinformatics Technology Co., Ltd. (Beijing, China). Genomic DNA samples were fragmented using the Covaris ultrasonic disruptor (Covaris Inc., Woburn, MA, USA), followed by the construction of a 350 bp short fragment library. Sequencing was performed on the Illumina NovaSeq PE150 platform (Illumina, San Diego, CA, USA). The raw data were processed using genome assembly tools including SOAP denovo, SPAdes and ABySS, and then was integrated with CISA. The assembly was further refined with GapCloser v1.12-ro, filtering out fragments below 500 bp, resulting in a draft genomic scaffold sequence. GeneMarkS 4.17 was employed for the prediction of coding genes. Subsequently, the protein sequences of structural genes were functionally annotated through Diamond alignment against universal databases including GO (Gene Ontology), KEGG (Kyoto Encyclopedia of Genes and Genomes), COG (Clusters of Orthologous Groups), NR (Non-Redundant Protein Database), Pfam, TCDB (Transporter Classification Database), and Swiss-Prot [32,33,34,35,36]. The alignment results with the highest score (minimum identity ≥ 40%, coverage ≥ 40%) were selected for annotation. The critical quality control metrics for the Lp20 genome assembly are detailed in Table S2.

Genome comparisons were conducted using BLAST v2.11.0. Circular genome maps were generated using the BRIG v0.95 JAVA script based on CGView [37]. Lp20 and WCFS1 were compared using SnapGene 6.0.2 to assess genomic structure, functional content, and similarity metrics such as ANI v1.34 and OrthoMCL v2.0.9, with differences evaluated based on unique genes, repeat elements, and divergence in virulence or antibiotic resistance genes.

### 2.4. Construction of bsh1 Deletion Mutant and Complemented Strains

The entire open reading frame of the *bsh1* gene was deleted by the homologous double-overcrossing recombination. A schematic diagram of the suicide plasmid construction and *bsh1* deletion is shown in Figures S2 and S3. Primer pairs used in this study are listed in Table S3. Homologous arms were amplified using two primers pairs (Lp20_GM1654L-F/Lp20_GM1654L-R and Lp20_GM1654R-F/Lp20_GM1654R-R), and then cloned into pUC19E by seamless cloning (CWBIO, Beijing, China) to obtain pUCbsh1. This suicide plasmid was then transformed into *L. plantarum* Lp20 cells by electroporation. MRS agar plates containing erythromycin were used to screen for positive colonies, which were then continuous cultivation in antibiotic-free MRS for 20 passages to select for double-overcrossing transformants. The replica plating method was performed to screen for colonies sensitive to erythromycin, and meanwhile hydrolase precipitation zones of this positive colonies disappeared (Figure S4C). Subsequently, the *Δbsh1* gene knockout mutants were confirmed by PCR with primers pairs Up-F/Up-R (UF/UR) and Down-F/Down/R (DF/DR).

To generate a gene complementation, the *bsh1* gene from *L. plantarum* Lp20 was amplified with primers (NcoI-bsh1/SacI-bsh1) and then cloned into pNZ11. Then, the recombinant pNZ*bsh1* plasmid was electroporated into the *L. plantarum* Lp20-*Δbsh1* gene knockout mutants to obtain the complementation strain, which was designated as *L. plantarum* Lp20-*Δbsh1*^+^.

### 2.5. Animal Experiment

Specific pathogen-free C57BL/6J mice (male, 6–8 weeks) were purchased from Beijing Huafukang Bioscience Co., Ltd. (Beijing, China) and maintained in barrier facility conditions (22 ± 2 °C, 50 ± 5% humidity, 12-h light/dark cycle and free access to food and water). Chew sticks were placed in each cage to enrich the environmental conditions. Following a 7-day acclimatization period, animals were randomly assigned to 4 groups using a computer-generated random number sequence: the low-fat diet group (LFD, *n* = 6) was fed a normal diet with 10% of energy from fat (D12450J, Research Diets, Table 1) for 12 weeks; the HFD groups were given a diet with 60% of energy from fat (D12492, Research Diets, Table 1) for 12 weeks to induce obesity (HFD, *n* = 6). The successful establishment of the obesity model was defined as a twenty percent increase in the mean body weight of all HFD-fed mice compared to that of LFD group [38]. Subsequently, obese mice were treated with bacteria strains (*n* = 6): (1) HFD group, fed with HFD and PBS; (2) HFD + Lp20-WT group, fed with HFD and 1 × 10^9^ CFU wild-type *L. plantarum* Lp20 with BSH activity; (3) HFD + Lp20-*Δbsh1* group, fed with HFD and 1 × 10^9^ CFU *L. plantarum* Lp20-*Δbsh1* mutant. PBS or bacterial suspension was daily administered by oral gavage at a volume 0.2 mL for additional 8 weeks.

Body weight of each group mice was recorded weekly, and the average daily energy intake was calculated according to energy density of diets in Table 1 (Tables S4 and S5). The experimental endpoint was defined when the body weight of mice in the HFD group was significantly higher than that in the intervention group and remained stable for 2 weeks. Before sacrificing, mice have been fasted overnight, anesthetized with isoflurane (Shenzhen RWD Life Science Co., Ltd., Shenzhen, China). Body composition, including fat mass and lean mass, was analyzed before sacrifice using a MiniQMR23-060H-I type Body Composition Analyzer (Shanghai Niumag Corporation, Shanghai, China). Blood serum was collected after euthanasia by cervical dislocation. Organs and adipose tissues, including epididymal white adipose tissue (eWAT), inguinal white adipose tissue (iWAT), and mesenteric fat were collected and weighed immediately. The experimental design was evaluated and approved by the Institutional Animal Care and Use Committee of the China Agricultural University, Beijing, China (protocol code AW81503202-5-2 and 18 May 2023 of approval). All procedures were performed in accordance with the National Research Council’s Guide for the Care and Use of Laboratory Animals.

### 2.6. Biochemical Assay of Blood Serum

We measured key indicators of metabolic status and liver function in the blood, including total cholesterol (TC), high-density lipoprotein cholesterol (HDL-C), low-density lipoprotein cholesterol (LDL-C), alanine aminotransferase (ALT), and aspartate aminotransferase (AST). The measurements were performed using a Hitachi Automatic Analyzer 3100 (Hitachi, Tokyo, Japan), with reagents from a biomedical analysis kit supplied by Maccura Biotechnology Co., Ltd. (Chengdu, China).

### 2.7. Liver Histological Analysis

Liver tissues were fixed in 4% paraformaldehyde, followed by progressively dehydrated and embedded in paraffin wax. Then, the liver tissues were cut into 4 μm sections and stained with hematoxylin and eosin (H&E) and periodic acid-Schiff (PAS). Histological and histochemical assessments were conducted in a blinded fashion. The scoring was performed according to the criteria established by Tian et al. [39].

### 2.8. RT-qPCR

The total RNA of mice liver tissues was extracted by AG RNAex Pro reagent (Accurate Biotechnology Hunan Co., Ltd., Changsha, China). The A260/280 ratios of all samples were between 1.8 and 2.1, indicating high RNA integrity and purity. For reverse transcription, we utilized 1 μg of the total RNA, combined with the reverse transcription kit designated for qPCR. RT-qPCR was then performed using the SYBR Green Premix Pro Taq Hs qPCR kit (Accurate Biotechnology Hunan Co., Ltd., Changsha, China) with QuantStudio 5 Real-Time PCR Systems (Thermo Fisher, Wilmington, NC, USA). All the primers used in this study are presented in Table 2. The amplification efficiency of all primers was validated using standard curve experiments. The relative quantification of the target gene is determined, and *β-actin* was used as the reference, in accordance with the 2^−ΔΔCT^method [40]. β-actin exhibited stable expression in liver tissue samples under experimental conditions.

### 2.9. Microbiome Analysis

Fecal samples were processed for gut microbiome profiling (*n* = 4). DNA was extracted using the PF Mag-Bind Stool DNA Kit (Omega, GA, USA), and the V3–V4 region of the 16S rRNA gene was amplified with barcoded primers (338F/806R). Amplicons were purified, pooled, and sequenced (2 × 300 bp) on Illumina MiSeq (Illumina, San Diego, CA, USA) [41]. Quality control of the raw paired-end sequencing data was performed using fastp v0.19.6 [42]. Subsequently, paired-end reads were merged into single sequences via FLASH v1.2.11 [43], with an overlap length of at least 10 bp and a maximum mismatch rate of 0.2 in the overlapping regions. Then, the optimized sequences, obtained following quality control and merging, were subjected to denoising using the DADA2 [44] or Deblur [45] plugin in the Qiime2 pipeline, with all default parameters. Finally, taxonomic assignment of the resulting amplicon sequence variants (ASVs) was performed using the Naive Bayes, Vsearch, or Blast classifier in Qiime2, against the Silva 16S rRNA gene database (v138). All data analyses were conducted via the Majorbio Cloud Platform (https://cloud.majorbio.com, accessed on 15 June 2025). α-diversity differences were assessed using the Kruskal-Wallis test, β-diversity differences between groups were evaluated with ANOSIM, and species-level differences were identified using the Wilcoxon test. The 16S analyses was conducted in a blinded manner. Specifically, the personnel performing the 16S analysis were blinded to the sample groups during the data acquisition and analysis process. The raw sequencing data generated in this study have been deposited in the National Center for Biotechnology Information (NCBI) database; the 16S rRNA gene sequencing accession number is PRJNA1358044 (https://dataview.ncbi.nlm.nih.gov/object/PRJNA1358044, accessed on 1 November 2025).

### 2.10. Statistical Analysis

Statistical analyses were conducted using GraphPad Prism version 9.0 (GraphPad, San Diego, CA, USA). Data were verified for normality using the D’Agostino and Pearson omnibus normality test. The difference in hydrolase activity between wild-type and mutant strains was assessed using Student’s *t*-test. All other data were analyzed using one-way ANOVA followed by Dunnett’s multiple comparison test, with LFD as control. Data are expressed as mean ± SEM. *p*-values < 0.05 were considered to be statistically significant.

## 3. Results

### 3.1. L. plantarum Lp20 Exhibited the Highest GDCA Hydrolase Activity

**Figure 1:**
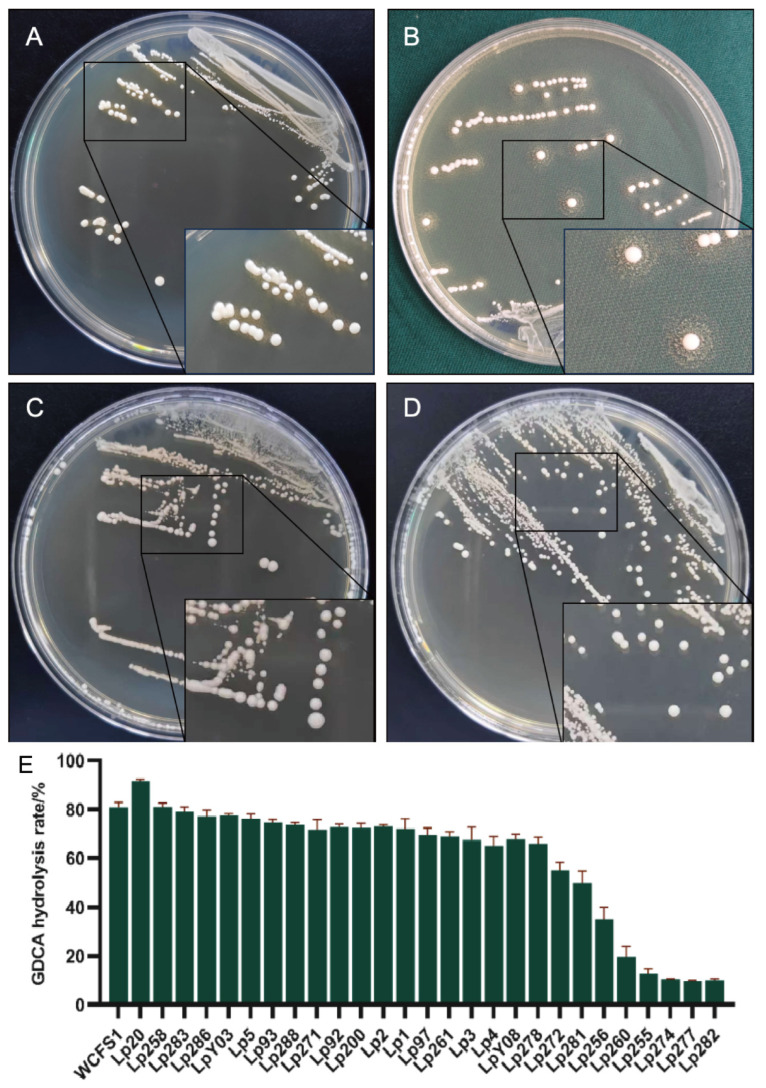
Assessment of BSH activity. (**A**,**B**) Precipitation halos around *L. plantarum* WCFS1 (**A**) and *L. plantarum* Lp20 (**B**) colonies on MRS-GDCA plates. (**C**,**D**) No precipitation halos around WCFS1 (**C**) and *L. plantarum* Lp20 (**D**) on MRS-TDCA plates. (**E**) BSH activities of twenty-eight *Lactiplantibacillus plantarum* strains toward GDCA.

Twenty-eight *L. plantarum* strains were investigated to produce BSH activity by bile salt plate method. Of these strains, 23 were able to produce precipitation halos on GDCA plates. Notably, *L. plantarum* Lp20 produced the most prominent precipitation halos on GDCA plate. The reference strain *L. plantarum* WCFS1 only produced white colonies with opaque granular precipitate halos (Figure 1A,B). However, none of these strains formed precipitation halos on TDCA plates; both the reference strain WCFS1 and *L. plantarum* Lp20 formed only uniform white colonies, indicating an absence of detectable BSH activity toward taurine-conjugated bile salts (Figure 1C,D).

The BSH activity of 28 *L. plantarum* strains was further measured using the ninhydrin assay. Most results were consistent with those of plate assay. *L. plantarum* Lp20 exhibited the highest GDCA hydrolysis activity, with a hydrolysis rate of 91.62% (Figure 1E). In addition, *L. plantarum* Lp255, 260, 274, 277, and 282 produced no deconjugation precipitation halos and showed GDCA hydrolysis rate below 15% (Figure S1). The bile salt plate method proves to be an effective and rapid screening method for identifying BSH-active strains. Consequently, *L. plantarum* Lp20 was selected to investigate the correlation between its BSH activity and the presence of BSH genes.

### 3.2. BSH-like Genes Were Located in the Chromosome of L. plantarum Lp20

**Figure 2:**
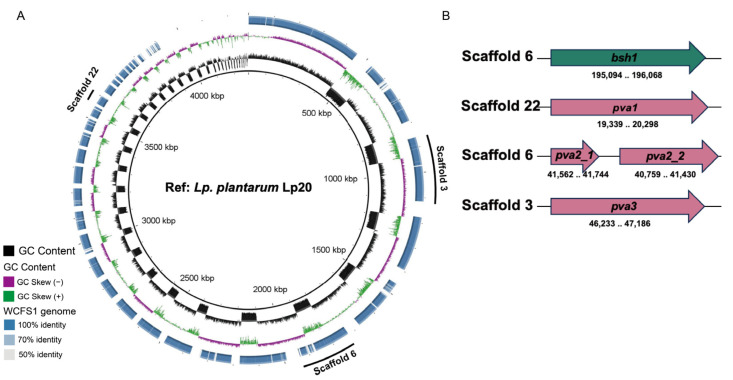
CGH genes were located in chromosome of *L. plantarum* Lp20 by comparing genomes with *L. plantarum* WCFS1. (**A**) Comparative genomic analysis of *L. plantarum* Lp20 strains based on BLAST_VERSION1 bin analysis. Matches with <50% identity or regions with no BLAST matches appear as blank spaces in the outer ring. The inner circle represents the reference sequence of *L. plantarum* Lp20. Ring of outer strain is *L. plantarum* WCFS1. (**B**) Schematic diagram of *L. plantarum* Lp20 cholylglycine hydrolase.

Comparison of cholylglycine hydrolase family genes of *L. plantarum* Lp20 with those in the *L. plantarum* WCFS1 genome using Clustal Omega alignment (Figure 2A and Figure S2). Draft genome sequencing demonstrated that *L. plantarum* Lp20 contains 4 choloylglycine hydrolase (CGH) family genes, identified as *bsh1*, *pva1*, *pva2* and *pva3* (Figure 2B). However, *pva2* gene appears to be a pseudogene due to a frame-shift at 109 bp downstream the start codon in *L. plantarum* Lp20. When compared to the amino acid sequences of Bsh1, Pva1 and Pva3 in *L. plantarum* WCFS1, the corresponding sequences in *L. plantarum* Lp20 showed similarities of 99.69%, 99.69%, and 97.48%, respectively. It was found that *pva2* and *pva4* were conservative for penicillin acylase activity, while *bsh1* contributed the major BSH activity in *L. plantarum* WCFS1 [27,46]. Therefore, the high BSH activity in *L. plantarum* Lp20 might be attributed to the gene *bsh1*.

### 3.3. Bsh1 Played a Key Role in the GDCA Hydrolysis Activity of L. plantarum Lp20

**Figure 3:**
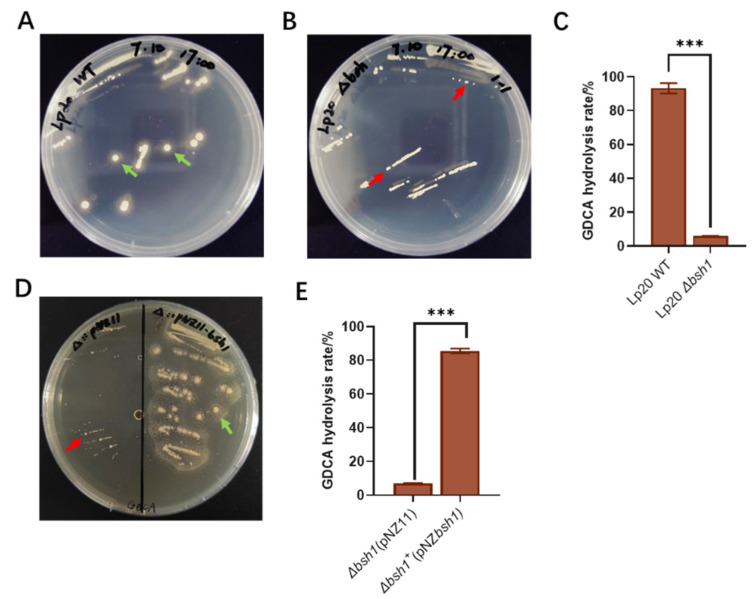
Identification of hydrolase activity of wild type and mutant strains. Bile salt agar precipitation halos assay of *L. plantarum* Lp20 wild type strain (**A**) and *L. plantarum* Lp20*-Δbsh1* strain (**B**). (**C**) GDCA hydrolysis rate of *L. plantarum* Lp20 and its *bsh1* mutant strain *L. plantarum* Lp20*-Δbsh1*. (**D**) *Bsh1* complementation strain *L. plantarum* Lp20*-Δbsh1^+^.* (**E**) GDCA hydrolysis rate of bile salt in the *L. plantarum* Lp20-*Δbsh1* and *L. plantarum* Lp20-*Δbsh1*^+^ strains complemented with pNZ11 (as control) and pNZbsh1 vector. Data are reported as the mean ± SEM from three independent experiments (*** *p* < 0.001). Red arrows denote the absence of bile salt agar precipitation halos; green arrows denote the presence of bile salt agar precipitation halos.

The *bsh1*-deleted strain, *L. plantarum* Lp20-*Δbsh1*, and complementary strain, *L. plantarum* Lp20-*Δbsh1^+^*, were constructed in this study (Figures S3 and S4). The results revealed the absence of precipitation halo formation in the *L. plantarum* Lp20-*Δbsh1* mutant on GDCA plate (Figure 3A,B). Meanwhile, the rate of bile salt hydrolysis was only 5.86% (Figure 3C). The complementation of the *bsh1* gene into the *L. plantarum* Lp20-*Δbsh1* strain restored its hydrolysis activity (Figure 3D,E). These results confirm that the bsh1 gene confers GDCA hydrolysis activity to *L. plantarum* Lp20. Subsequently, *L. plantarum* Lp20 and *Δbsh1* mutant strain were used to investigate the relationship between BSH activity and obesity amelioration. To address the concern of potential differences in bacterial growth states, we compared the growth of the wild-type (WT) and Δbsh1 strains in MRS broth. The growth curves indicated that all strains exhibited comparable growth rates and biomass production (Figure S5).

### 3.4. L. plantarum Lp20 Decreased Weight Gain and Fat Accumulation in Obese Mice

**Figure 4:**
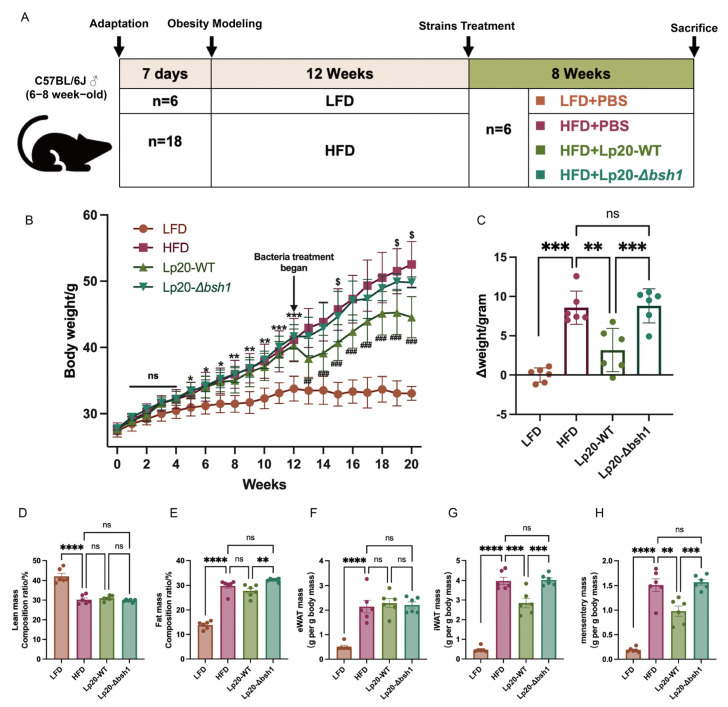
Effects of Lp20-WT and Lp20-*Δbsh1* on bodyweight and adipose tissue mass analysis in mice. (**A**) Animal experiment design. (**B**,**C**) Body weight change (**B**) and net body weight gain (**C**) of four treatment groups. (**D**–**H**) Body composition analysis (**D**,**E**) and adipose tissue mass analysis including eWAT (**F**), iWAT (**G**) and mesenteric fat mass (**H**). Values are expressed as mean ± SEM (*n* = 6). ns, no significant difference; * *p* < 0.05, ** *p* < 0.01, *** *p* < 0.001 and **** *p* < 0.0001 represent differences between HFD-induced obese mice and LFD-fed mice during 1–12 weeks. ^##^ *p* < 0.01 and ^###^ *p* < 0.001 represent differences between HFD group and Lp20-WT group. ^$^ *p* < 0.05 represents difference between Lp20-WT and Lp20-*Δbsh1* group.

The obesity induced by high fat diet in mice and the grouping intervention scheme are shown in Figure 4A. The bodyweight of HFD group mice reached 41.03 g after 12 weeks, which was 1.21-fold higher than that of LFD group mice, indicating the successful establishment of an obese mouse model (Figure 4B). After 8 weeks *L. plantarum* Lp20 intervention, the net body weight gains of mice in the HFD, Lp20-WT, and Lp20-Δbsh1 groups averaged 8.58 g, 3.18 g, and 8.81 g, respectively (Figure 4C). This finding, when combined with the analysis of energy intake data from each group (Table S6), suggests that Lp20 with BSH enzyme activity can significantly reduce weight gain in a manner that appears unrelated to energy intake. Moreover, *L. plantarum* Lp20 treatment decreased fat mass ratio in body composition compared with the *L. plantarum* Lp20-*Δbsh1* mutant treatment group, without affecting the lean mass (Figure 4D,E). Furthermore, no difference in eWAT mass was observed among the three groups; however, iWAT mass and mesenteric fat mass were significantly lower in the Lp20-WT group than in both the Lp20-*Δbsh1* and HFD groups (*p* < 0.01) (Figure 4F–H). However, no difference was observed between the Lp20-*Δbsh1* group and HFD group. Taken together, the bile salt hydrolase *bsh1* of *L. plantarum* Lp20 contributes to ameliorating obese-related syndromes by reducing weight gain and body fat accumulation.

### 3.5. Bsh1 Alleviated Liver Steatosis and Decreased Serum Cholesterol in Obese Mice

**Figure 5:**
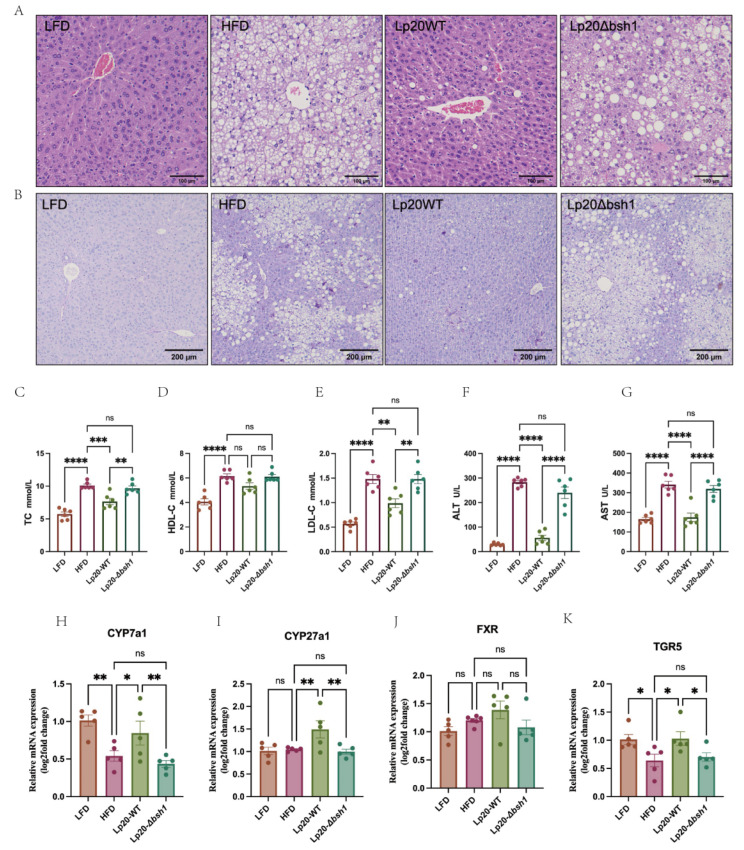
The impact of a HFD and *L. plantarum* Lp20 bile salt hydrolase on liver steatosis and serum bio-markers levels. H&E (**A**) and PAS (**B**) stained liver sections from LFD, HFD, Lp20-WT, and Lp20-*Δbsh1* group. (**C**–**E**) represent serum TC, high-density lipoprotein cholesterol, and LDL-C, respectively. (**F**) Serum alanine aminotransferase. (**G**) Serum AST. Values are expressed as mean ± SEM (*n* = 6). (**H**–**K**) The mRNA expression of *CYP7a1*, *CYP27a1*, *FXR*, *TGR5* in mice liver (*n* = 5). ns, no significant difference; * *p* < 0.05, ** *p* < 0.01, *** *p* < 0.001 and **** *p* < 0.0001 showed the significant difference with ANOVA analysis.

We conducted a comprehensive histopathological analysis of liver tissues. In this study, livers from the HFD and Lp20-*Δbsh1* groups appeared pale yellow, indicative of hepatic steatosis. By contrast, healthier, slightly redder livers were observed in the Lp20-WT and LFD groups (Figure S6). Liver weights were 1.97 ± 0.23 g in the HFD group and 1.20 ± 0.19 g in the Lp20-WT group, the latter being significantly lower than in the Lp20-*Δbsh1* group (Table S10). Additionally, there was no significant difference in liver weight between the Lp20-WT group and LFD group. Collectively, these findings indicate that the BSH activity of Lp20 exerts a protective effect against host liver injury. Hepatocytes in LFD mice displayed purple-red cytoplasmic staining with uniformly stained chromatin, indicating negligible fat accumulation and the absence of steatosis (Figure 5A). However, vacuolization due to lipid accumulation was observed in the livers of HFD mice. NAS scores revealed that Lp20 administration significantly reduced HFD-induced histological damage (Figure S7). PAS staining corroborated these results by distinguishing steatosis from glycogen or other polysaccharide accumulation (Figure 5B). It is worth noting that the Lp20-WT group exhibited a darker cytoplasm and a substantial reduction in lipid droplets. However, the Lp20-*Δbsh1* group showed steatosis comparable to that of the HFD group in both droplet size and number.

Serum cholesterol levels were subsequently quantified for all experimental groups. After 8 weeks of feeding with the HFD, the TC levels of the HFD group increased to 9.15 mmol/L, which was 1.76-fold higher than that of the LFD group. Notably, only Lp20-WT treatment significantly reduced the total serum cholesterol compared with the HFD group, while no significant difference was observed between the mutant and HFD groups (Figure 5C). Further analysis revealed that Lp20-WT primarily decreased serum LDL-C levels, with no significant impact on serum HDL-C levels (Figure 5D,E). Therefore, the bile salt hydrolase Bsh1 of the *Lp. plantarum* Lp20 appears to play a role in mitigating abnormal blood lipid levels by regulating serum cholesterol levels in obese mice. Furthermore, compared with the Lp20-*Δbsh1* group, the Lp20 group exhibited reductions in serum ALT and AST of 76.6% and 45.3% (Figure 5F,G).

To further investigate the hepatic bile acid synthetic pathways, we quantified key transcripts related to bile acid synthesis using RT-qPCR. Compared with the Lp20-*Δbsh1* group, the Lp20-WT group exhibited a marked upregulation of *CYP7a1* and *CYP27a1*, which encode the rate-limiting enzymes in the classical and alternative bile acid biosynthetic pathways, respectively (Figure 5H,I). Additionally, Lp20-WT significantly increased the hepatic expression of the bile acid-activated receptors *FXR* and *TGR5* (*p* < 0.05, Figure 5J,K). Collectively, these findings suggest that bacterial activity dependent on BSH within the gut microbiota may transcriptionally modulate hepatic bile acid synthesis and sensing pathways under diet-induced obesity.

### 3.6. L. plantarum Lp20 Restored the Intestinal Microbial Community

**Figure 6:**
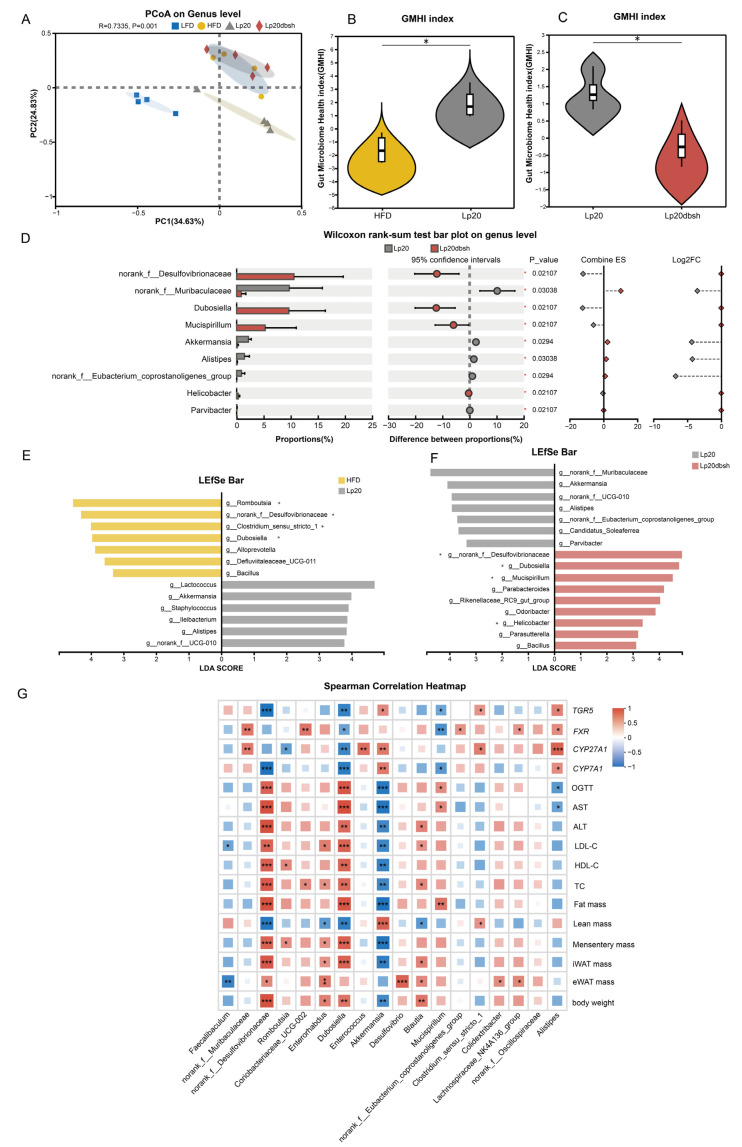
Variation of fecal microbiota composition in response to *L. plantarum* Lp20 intervention. (**A**) PCoA of the community structure. (**B**,**C**) Gut Microbiota Health Index at the genus level. (**D**) Differential species testing at the genus level. (**E**,**F**) Linear discriminant analysis effect size (LEfSe). (**G**) Heat map depicting Spearman correlations between fecal microbial ASV abundances (16S rRNA sequencing) and host metabolic phenotypes. * *p* < 0.05, ** *p* < 0.01, *** *p* < 0.001; *n* = 4 mice per group.

To systematically evaluate the therapeutic potential of Lp20-WT in reversing HFD-induced dysbiosis, we profiled faecal microbial communities by 16S rRNA gene sequencing. The analysis of the Shannon index revealed a significant increase in α-diversity within the Lp20-WT group relative to the LFD group (Figure S8). Moreover, when compared to the LFD group, the HFD group exhibited an augmented gut microbiota diversity, which may be associated with an increased fiber intake [47]. β-diversity principal-coordinate analysis (PCoA, Bray–Curtis distance) revealed complete segregation of LFD and HFD groups along PC1, indicating that HFD profoundly reshaped gut microbial architecture. Strikingly, Lp20-WT intervention steered the microbial community away from the HFD-associated configuration, indicating a partial restoration of both richness and composition, whereas the Lp20-Δbsh1 cohort remained indistinguishable from the HFD group (Figure 6A). Quantitation using the Microbiota Health Index (MHI) corroborated these findings: Lp20-WT fully restored HFD-lowered MHI scores (*p* < 0.05), whereas MHI values in the Lp20-Δbsh1 group remained indistinguishable from those of the HFD cohort (Figure 6B,C). Lp20-WT expanded beneficial SCFA producers—including *norank_f__Muribaculaceae*, *Akkermansia*, and *Alistipes*. At the same time, it suppressed LPS-producing pro-inflammatory taxa such as *norank_f__Desulfovibrionaceae*, *Dubosiella*, and *Mucispirillum* (Figure 6D). LEfSe with LDA score > 3.0 further disclosed that the HFD microbiota was dominated by potential pathogens (*g__Romboutsia*, *g__norank_f__Desulfovibrionaceae*, and *g__Dubosiella*). In contrast, Lp20-WT fostered a beneficial consortium centered on *Akkermansia* and *Alistipes*, whereas Lp20-*Δbsh1*-treated mice retained a pro-inflammatory signature enriched for *g__norank_f__Desulfovibrionaceae*, *g__Dubosiella*, and *g__Mucispirillum* (Figure 6E,F), reinforcing that loss of BSH activity attenuates probiotic efficacy. Furthermore, we analyzed the correlation between bacterial abundance and host phenotypes (Figure 6G). The relative abundance of fecal *Akkermansia* was significantly negatively correlated with white adipose tissue mass and serum levels of TC, ALT and AST, but positively correlated with lean mass and the hepatic expression of BA synthesis rate-limiting enzymes *CYP7A1* and *CYP27A1*, as well as nuclear receptors *FXR* and *TGR5*. In contrast, the abundance of *norank_f__Desulfovibrionaceae* and *Dubosiella* was positively associated with metabolic inflammatory phenotypes. These results collectively demonstrate that Lp20-WT improves gut microbial balance through BSH-dependent metabolic regulation, thereby restoring host metabolic homeostasis.

## 4. Discussion

The global shift toward high-fat, high-calorie diets, fueled by economic growth, has led to a surge in obesity, a key risk factor for type 2 diabetes, atherosclerosis, and related mortality [48]. Conventional interventions are hindered by poor adherence, gastrointestinal side effects, and high costs. In response, probiotic interventions are emerging as a promising new strategy for managing metabolic disorders [49].

Accumulating evidence demonstrates that BSH acts as a metabolic gatekeeper, promoting the conversion of primary BAs into secondary BAs. Upon microbial deconjugation of GDCA, the secondary bile acid DCA is released into the intestinal lumen and functions as a selective signaling metabolite. It engages the bile-acid-sensing receptors FXR and TGR5 expressed in skeletal muscle, brown adipose tissue, and enteroendocrine L-cells, thereby initiating downstream transcriptional cascades that systemically modulate lipid and energy homeostasis [12,13]. A recent study indicates that BSH/T enzymes from Bifidobacterium animalis can extend their catalytic activity beyond classic deconjugation, linking tryptophan with bile acids to form Trp-CA, a novel microbial-derived bile acid that boosts GLP-1 release and insulin sensitivity [50]. Moreover, these enzymes exhibit acyltransferase activity [51]. Such strain-specific remodeling of the bile acid pool is likely to influence microbial fitness within the intestinal niche, underscoring the need for mechanistic studies that dissect its ecological and metabolic consequences.

The BSH activity of different lactic acid bacteria strains varies significantly, with the genus *Lactiplantibacillus* serving as a major microbial reservoir of this enzyme [52]. Here we found that most *L. plantarum* strains have a strong specificity to glycine-conjugated bile salts, which is likely because *L. plantarum* is one of the prevalent strains in the human intestinal microbiota. The existence of BSH activity is considered a conservative microbial adaptation to the human intestinal environment [53,54]. Given that more than 60% of BAs in the human bile acid pool are glycine-conjugated, the general deconjugating activity and preference for glycine-conjugated bile salts by *Lactiplantibacillus* likely result from the evolutionary conservation of the BSH gene [55,56]. In this study, we screened *L. plantarum* Lp20 for its exceptionally high BSH activity and engineered an isogenic *Δbsh1* mutant that lost > 94% of GDCA hydrolysis capacity—an observation that aligns with prior work in *L. plantarum* WCFS1 [46]. To establish a causal link between *bsh1* and host energy balance, diet-induced obese mice were randomly assigned to receive either the Lp20-WT or the Lp20-*Δbsh1* variant by daily gavage for 8 consecutive weeks.

In vivo experiments demonstrated that daily gavage of *L. plantarum* Lp20 markedly reduced body weight, white-adipose-tissue mass, serum TC and LDL-cholesterol relative to high-fat-diet (HFD) controls; moreover, the Lp20 modulated gut microbiota composition by expanding beneficial taxa while suppressing potential pathogens, thereby alleviating diet-induced obesity. Strikingly, these metabolic benefits were almost completely abolished in mice receiving the isogenic Lp20-*Δbsh1*. Notably, HFD-induced liver steatosis has been reported in previous studies [57,58]. Increased ALT and AST levels in the bloodstream are typically indicative of liver injury or steatosis [59]. However, there are few studies which confirmed the anti-liver steatosis effect of Bsh1 in *Lactobacillus* strains. The reduction in serum ALT and AST levels and the histopathological improvements observed through H&E and PAS staining provide strong evidence for the liver-protective role of *bsh1* from Lp20. Consequently, these results indicate that Lp20-WT significantly alleviates liver lipid accumulation, suggesting a potential therapeutic benefit of BSH in Non-Alcoholic Fatty Liver Disease (NAFLD). Therefore, it can be inferred that BSH activity might play a protective role against liver injury. When BAs are deconjugated by BSH, the solubility of bile salts decreases, resulting in an increased excretion of BAs through feces due to their impaired reabsorption in the enterohepatic circulation. This reduction in reabsorption capacity subsequently diminishes the small intestine’s ability to absorb lipids from chylomicrons. Concurrently, to compensate for the loss of BAs, the liver is prompted to utilize cholesterol for the synthesis of new BAs, thereby lowering serum cholesterol levels [24]. In this study, we observed that the upregulation of the rate-limiting bile acid synthesis genes, CYP7A1 and CYP27A1, suggests that Lp20 intervention promotes the utilization of hepatic cholesterol for bile acid resynthesis, thereby reducing serum cholesterol levels. Simultaneously, increased expression of hepatic farnesoid X receptor (FXR) was noted. This may stem from the fact that upon activation, hepatic FXR predominantly suppresses CYP8B1, with minimal effects on CYP7A1 [60]. The activation of hepatic FXR could modulate triglyceride synthesis and mitigate hepatic steatosis through alternative signaling pathways, though the precise mechanisms warrant further investigation. Moreover, the enterohepatic circulation of secondary BAs via the portal vein may enhance the activation of receptors such as TGR5, leading to reduced lipid synthesis and elevated carbohydrate metabolism [25,61]. Consistently, our study revealed increased hepatic TGR5 expression, implying that bile acid signaling transcriptional regulation contributes to Lp20’s anti-obesity effects.

By comparing the isogenic Δbsh1 knockout mutant with its wild-type counterpart, our study shows that BSH plays an essential role in the anti-obesity phenotype of *L. plantarum* Lp20. Notably, Lp20 may also produce other beneficial metabolites, such as short-chain fatty acids (SCFAs) and conjugated linoleic acid (CLA), which warrant further investigation in future studies. Our study has several limitations. The sample size was small (*n* = 6), which may affect the statistical power and the ability to generalize our findings. Only male mice were used, so the results may not apply to female mice. We did not perform dose-response testing, so the optimal dose of L. plantarum Lp20 for anti-obesity effects is unknown. Finally, we did not conduct bile acid profiling or validate in vivo metabolic endpoints, nor did we measure GLP-1 levels. Although L. plantarum Lp20 exhibits significant BSH activity and metabolic benefits, its mechanism of action remains to be further elucidated. Future research should focus on high-throughput screening of various lactobacilli and bifidobacteria strains to identify those with the highest BSH activity. Subsequent in vivo studies in animal models and human subjects should assess the metabolic effects of these strains, taking into account the distinct bile acid conjugation patterns and microbiome profiles between species. This will help determine their potential for clinical application in humans.

## 5. Conclusions

The BSH is recognized as a metabolic marker for probiotics with the ability to reduce lipid accumulation. In this study, *L. plantarum* Lp20 was screened for its highest BSH activity among 28 candidate strains. To establish a causal link between BSH and anti-obesity efficacy, we constructed a *bsh1* knock-out mutant; comparative analyses confirmed that the anti-obesity phenotype of Lp20 is BSH-dependent. In a diet-induced obese mouse model, the Lp20-WT administration markedly curbed weight gain, lowered serum TC, and reversed hepatic steatosis. Mechanistically, the Lp20-WT up-regulated the expression of the rate-limiting bile-acid-synthetic enzymes CYP7A1 and CYP27A1, activated the receptor FXR and TGR5, and thereby modulated bile-acid-related pathways. Concomitantly, Lp20-WT modulated microbiota composition and re-established micro-ecological homeostasis. In contrast, the Lp20-Δbsh1 group exhibited almost complete loss of these metabolic protective effects. These findings provide strong evidence in this model that BSH activity underlies the obesity-ameliorating effects of Lp20, while recognizing that additional microbial metabolites (e.g., SCFAs, CLA) may also participate.

In mice, bile acids are predominantly tauroconjugated, while in humans, they are primarily glycoconjugated; this difference might affect Lp20’s efficacy in humans. Also, the human gut microbiota differs from that of laboratory mice in both taxonomy and function. As a pre-clinical model, this study is limited by the small number of male mice used. Future studies should therefore validate these findings in female mouse, perform dose-response and long-term safety assessments, and ultimately conduct randomized placebo-controlled trials that monitor systemic bile-acid profiles, GLP-1 dynamics, and microbiome shifts to determine whether Lp20 BSH activity translates into clinically meaningful metabolic benefits in human obesity.

## Figures and Tables

**Table 1:** Dietary composition and energy density in animal experiments.

Product	D12450J (LFD)		D12492 (HFD) *	
	gm%	kcal%	gm%	kcal%
Protein	19.2	20.0	26	20.0
Carbohydrate	67.3	70.0	26	20.0
Fat	4.3	10.0	35	60.0
**Ingredient**	**gm**	**kcal**	**gm**	**kcal**
Casein, 30 Mesh	200	800	200	800
L-Cystine	3	12	3	12
Corn Starch	506.2	2024.8	0	0
Maltodextrin 10	125	500	125	500
Sucrose	68.8	275.2	68.8	275.2
Cellulose, BW 200	50	0	50	0
Soybean Oil	25	225	25	225
Lard	20	180	245	2205
Mineral Mix S10026	10	0	10	0
Dicalcium Phosphate	13	0	13	0
Calcium Carbonate	5.5	0	5.5	0
Potassium Citrate·1H_2_O	16.5	0	16.5	0
Vitamin Mix V10001	10	40	10	40
Choline Bitartrate	2	0	2	0
FD&C Yellow Dye #5	0.04	0	0	0
FD&C Red Dye #40	0	0	0	0
FD&C Blue Day #1	0.01	0	0.05	0
Total	1055.05	4057	773.85	4057
kcal/gm	3.85		5.24	

* As for D12492, the content of cholesterol is 279.6 mg/kg.

**Table 2:** Primers used in this study.

Gene	Primer Sequence (5′-3′)
*CYP7a1*	Forward: GGGATTGCTGTGGTAGTGAGC
	Reverse: GGTATGGAATCAACCCGTTGTC
*CYP27a1*	Forward: CCAGGCACAGGAGAGTACG
	Reverse: GGGCAAGTGCAGCACATAG
*FXR*	Forward: GCTTGATGTGCTACAAAAGCTG
	Reverse: CGTGGTGATGGTTGAATGTCC
*TGR5*	Forward: CCTGGCAAGCCTCATCGTC
	Reverse: AGCAGCCCGGCTAGTAGTAG
*β-actin*	Forward: GTGACGTTGACATCCGTAAAGA
	Reverse: GCCGGACTCATCGTACTCC

## Data Availability

All data presented in this study is available from the corresponding author upon reasonable request. The draft genome and assembly of *Lactiplantibacillus plantarum* Lp20 strain was deposited to GenBank (Accession number: JBAJNI000000000). The 16S rRNA gene sequencing accession number is PRJNA1358044 (https://dataview.ncbi.nlm.nih.gov/object/PRJNA1358044, accessed on 1 November 2025).

## Supplementary Materials

The following supporting information can be downloaded at: https://www.mdpi.com/article/10.3390/nu17223555/s1, Table S1: Bacteria strains and plasmids used in this study; Table S2: Key quality control metrics for the Lp20 genome assembly; Table S3: Primers used in this study; Table S4: Dietary composition and energy density in animal experiments; Table S5: Mice average daily energy intake during the modeling period (1–12 weeks); Table S6: Mice average daily energy intake during treatment (13–20 weeks); Table S7: Weight trajectories and Area Under the Curve (AUC) for individual mice during the modeling period (Weeks 1–12); Table S8: Weight trajectories and Area Under the Curve (AUC) for individual mice during treatment (13–20 weeks); Table S9: NAS scoring of NASH; Table S10: Liver weight and liver index (Mean and standard deviation) of mice after 8 weeks of intervention. Figure S1: Negative results in precipitation halo assay for Lactiplantibacillus BSH activity on GDCA plates; Figure S2: Comparison of cholylglycine hydrolase family genes of L. plantarum Lp20 with those in the L. plantarum WCFS1 genome using Clustal Omega alignment. Figure S3: A schematic representing the generation of a markerless bsh1 deletion in L. plantarum Lp20 using the pUCbsh1arm suicide plasmid; Figure S4: Illustrations of the PCR detection of bsh1 single crossover insertion vectors using primer pairs DF/UF and DR/UR; Figure S5: Growth curve measurement of wild-type and mutant strains; Figure S6: Fat distribution in abdominal cavity and schematic diagram of liver in mice; Figure S7: Hepatic NAS assessment; Figure S8: α-diversity of the gut microbiota, represented by Shannon index.
