# Supplementary material for: Lactiplantibacillus plantarum Lp20 Alleviates High Fat Diet-Induced Obesity in Mice via Its Bile Salt Hydrolase Activity
Source: Nutrients. 2025 Nov 14;17(22):3555. doi: 10.3390/nu17223555 (PMC12655260; doi:10.3390/nu17223555)
Supplement: Supplementary file 1 [file nutrients-17-03555-s001.zip › nutrients-3957003-supplementary.pdf]

Supplementary Material

Lactiplantibacillus plantarum Lp20 Alleviates High Fat Diet-Induced Obesity in Mice via Its Bile Salt Hydrolase Activity

Xiaoyue Bai [1], Fangzhou Lu [1], Yizhi Jing [2], Hui Wang [1], Haidong Qian [2], Ming Zhang [3], Zhengyuan Zhai [1,*] and Yanling Hao [2,*]

[1] College of Food Science and Nutritional Engineering, China Agricultural University, Beijing 100083, China; baixiaoyue21@163.com (X.B.); lufangzhou@smart.org.cn (F.L.); wanghui06@sanyuan.com.cn (H.W.)

[2] Key Laboratory of Precision Nutrition and Food Quality, Department of Nutrition and Health, China Agricultural University, Beijing 100190, China; j15502414985@163.com (Y.J.); qianhaidong2023@163.com (H.Q.)

[3] School of Food and Health, Beijing Technology and Business University, Beijing 100048, China; zhangming@th.btbu.edu.cn

* Correspondence: zhaizy@cau.edu.cn (Z.Z.); haoyl@cau.edu.cn (Y.H.); Tel.: +86-10-62737450 (Z.Z.); +86-13810369359 (Y.H.)

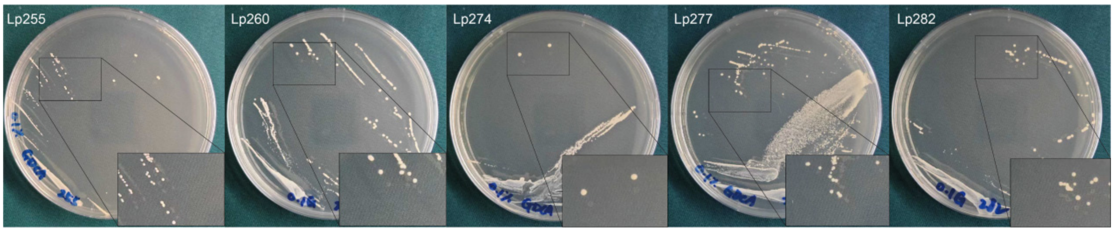

Figure S1. Assessment of BSH activity. Absence of precipitation halos around Lp255, Lp260, Lp274, Lp277, and Lp282 colonies on MRS-GDCA plates.

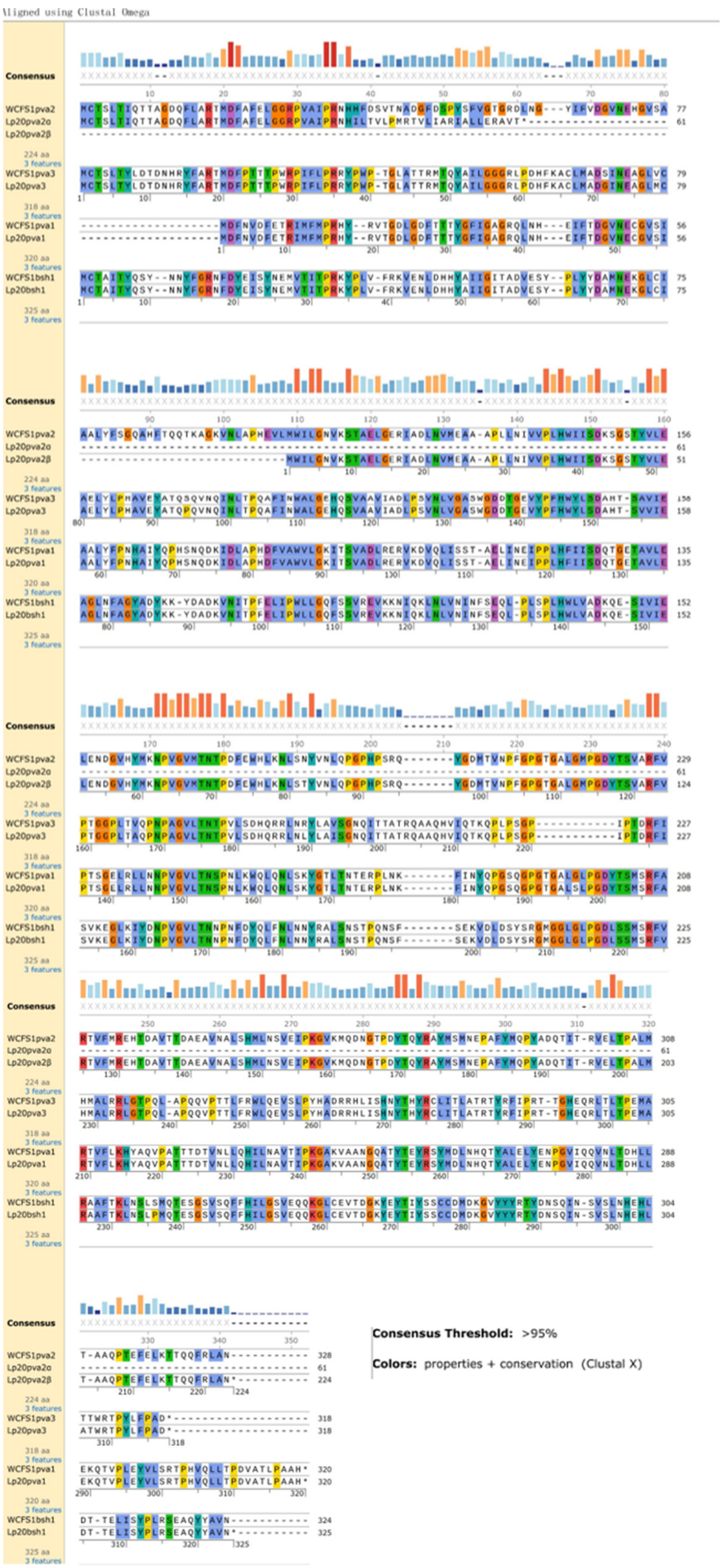

Figure S2. Comparison of cholylglycine hydrolase family genes of *L. plantarum* Lp20 with those in the *L. plantarum* WCFS1 genome using Clustal Omega alignment. The bar chart reflects the conservatism of the common sequence.

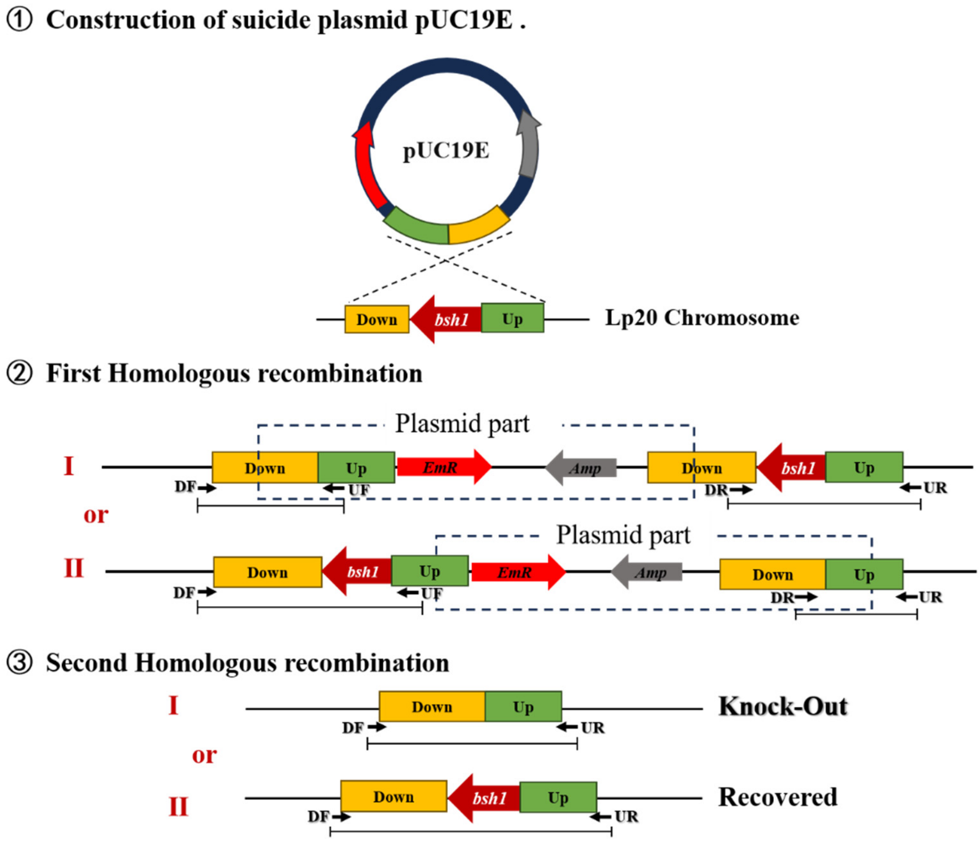

Figure S3. A schematic representing the generation of a markerless *bsh1* deletion in *L. plantarum* Lp20 using the pUCbsh1arm suicide plasmid. The process involves the following steps: (1) Construction of a suicide plasmid containing homologous flanking fragments of the *bsh1* gene. (2) Integration into the chromosome via the first recombination event in homologous recombination, with primer positions (DF/UF, DR/UR) used for plasmid insertion verification. (3) Selection of double crossover transformants by continuous cultivation in MRS medium without any antibiotics for 10 passages, and the use of primers (DF/UR) to differentiate the deletion mutants from the wild type.

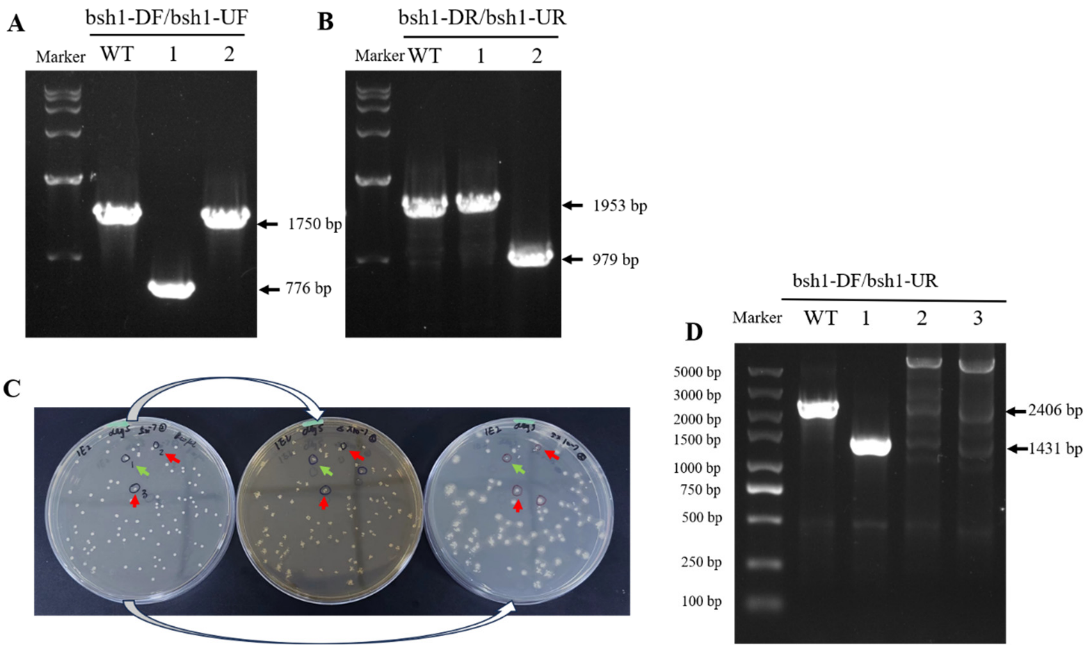

Figure S4. Illustrations of the PCR detection of *bsh1* single crossover insertion vectors using primer pairs DF/UF (A) and DR/ UR (B). Marker**,** DNA Ladder 15,000. WT, wild-type *L. plantarum* Lp20 genome used as a template; 1-2 are two single clones selected from the plates following electroporation. (C) depicts the single crossover strains after continuous passages, imprinted on antibiotic and GDCA media, selecting for bile salt hydrolase mutant single clones. The second homologous recombination mutants were PCR verified using the primer pair DF/UR in (D). 1-3 are three single clones chosen from the double selection imprinting plates as shown in (C) following the second homologous recombination. WT, wild-type strain *L. plantarum* Lp20. Marker, DNA Ladder 5,000.

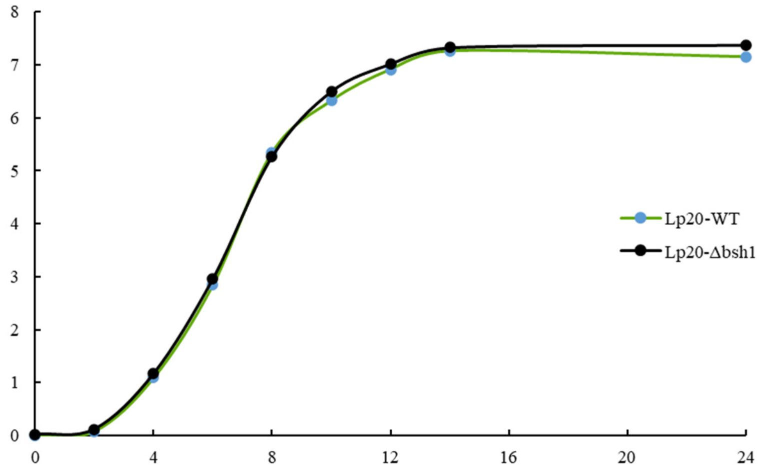

Figure S5. Growth curve measurement of wild-type and mutant strains.

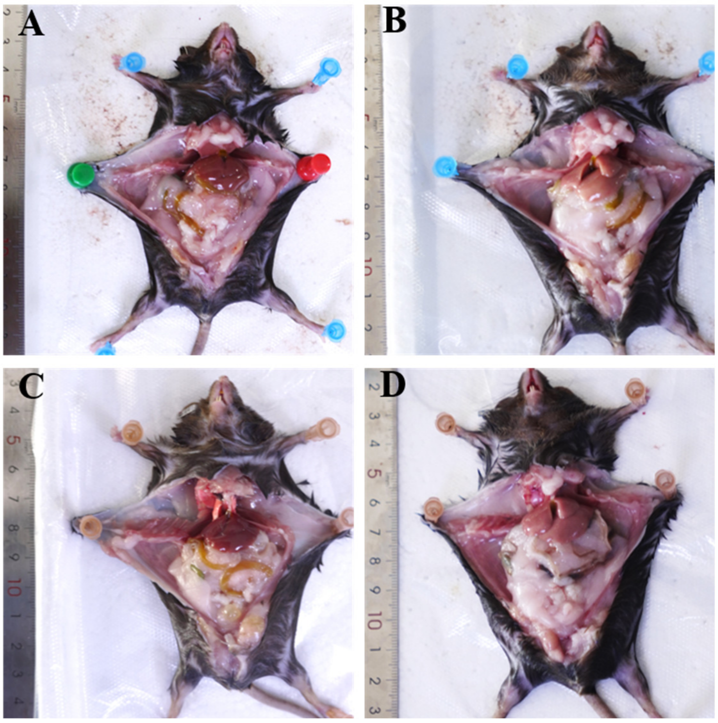

Figure S6. Fat distribution in abdominal cavity and schematic diagram of liver in mice. (A) LFD mice (B) HFD mice (C) HFD+Lp20-WT treated mice (D) HFD+ Lp20-*Δbsh1* treated mice.

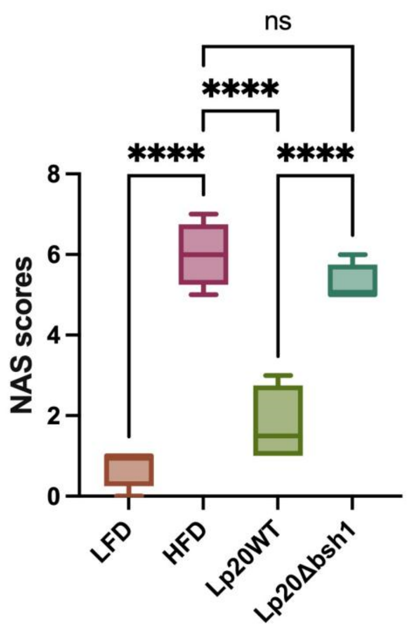

Figure S7. Hepatic NAS assessment.

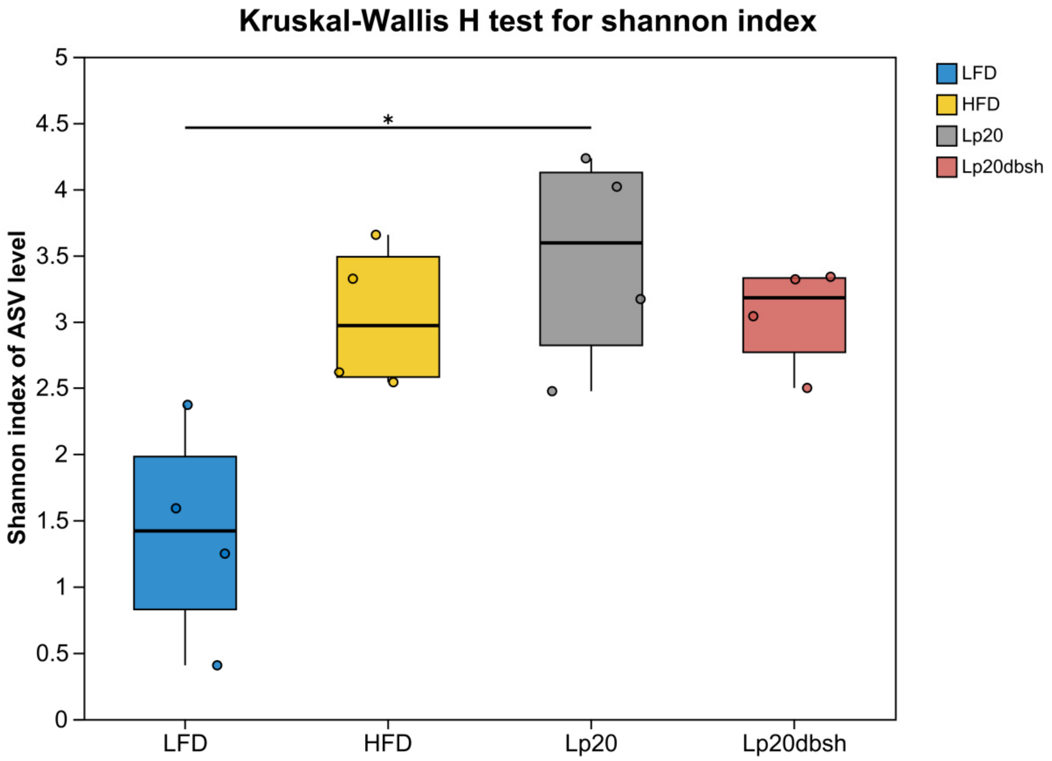

Figure S8. α-diversity of the gut microbiota, represented by Shannon index.

Table S1. Bacteria strains and plasmids used in this study.

Strains and plasmids	Relevant characteristics	Source or reference
**Strains**		
L. plantarum WCFS1	Standard strain of *Lactiplantibacillus plantarum*, used as control strain to determine bile salt clearance rate of BSH	Given by Professor William de Wos
L. plantarum Lp1, Lp2, Lp3, Lp4, Lp5, Lp92, Lp93, Lp97	Candidate strain	Isolated from fermented dairy products in Yunnan and preserved by our laboratory
L. plantarum Lp20	Candidate strain. Host strain for deletion mutant	Isolated from fermented dairy products in Yunnan and preserved by our laboratory
L. plantarum Y03, Y08	Candidate strain	Given by Professor Yang Yao
L. plantarum Lp200, Lp278, Lp281, Lp282, Lp283, Lp286, Lp288	Candidate strain	Laboratory collection
L. plantarum Lp255, Lp256	Candidate strain	Isolated from fermented food in Anhui and preserved by our laboratory
L. plantarum Lp258, Lp260	Candidate strain	Isolated from fermented dairy products in Inner Mongolia and preserved by our laboratory
L. plantarum Lp261	Candidate strain	Isolated from fermented dairy products in Gansu and preserved by our laboratory
L. plantarum Lp271	Candidate strain	Isolated from fermented dairy products in Qinghai and preserved by our laboratory
L. plantarum Lp272, Lp274	Candidate strain	Isolated from fermented Sauerkraut in Heilongjiang and preserved by our laboratory
L. plantarum Lp277	Candidate strain	Isolated from fermented products in Tianjin and preserved by our laboratory
L. lactic NZ9000	Plasmid-free derivative of *L. lactis* MG1363 *pepN*::*nisRK*. Host strain for pNZ vectors	Laboratory collection
E. coli DH5α	F^-φ80d*lac*ZΔM15, Δ (*lacZYA–argF*) U169, *deoR, recA1, endA1, hsdR17* (r_K^-, m_K^-), *phoA, supE44,* λ^-*, thi-1, gyrA96, relA1*. Host strain for pUC vectors	TIANGEN
Lp. plantarum Lp20 *Δbsh1*	*bsh1* gene deletion mutant of *Lp. plantarum* Lp20. Host strain for pNZ11, Cm^R.	This work
Lp. plantarum Lp20 *Δbsh1*	*Lp. plantarum* Lp20-*Δbsh1* with pNZ11 as control strain	This work
Lp. plantarum Lp20 *Δbsh1* $^+$	*Lp. plantarum* Lp20-*Δbsh1* with pNZbsh1	This work
**Plasmids**		

pUC19E	Suicide plasmid carried a *Em*^{R} cassette, derivative of pUC19, *Amp*^{R}, *Em*^{R}	Laboratory collection
pUCbsh1arm	pUC19E containing the homologous arms of *bsh1* gene of *Lp. plantarum* Lp20, *Amp*^{R}, *Em*^{R}	This work
pNZ11	pNZ8148 derivative carrying P11 promoter instead of PnisA promoter, *Cm*^{R}	Laboratory collection
pNZbsh1	pNZ11 containing the *bsh1* gene of *Lp. plantarum* Lp20, *Cm*^{R}	This work

Table S2. Key quality control metrics for the Lp20 genome assembly.

Metric	Value
**Coverage depth (×)**	25.55
**Scaffold N50 (bp)**	208,155
**GC content (%)**	44.56
**Genome completeness* (%)**	84.21
**Total assembly length (bp)**	3,156,820
**Number of scaffolds**	40
**Number of contigs**	41

Table S3. Primers used in this study.

Primer pair	Sequence (5'-3')
Lp20_GM1654L-F	aaaacgacggccagtgaattcGATCATGTTGTTAAACTCACTTACGATAA
Lp20_GM1654L-R	ggaggattactatttAAGCCACTACTGTAATAGTTAAAATTGTTT
Lp20_GM1654R-F	ggcttAAATAGTAATCCTCCTCGAAAATAATATTT
Lp20_GM1654R-R	cttctgcaggtcgactctagaCGGCCGACCGTAATATTGG
pUC antilinear-F pUC	AATTCACTGGCCGTCGTTTTACAAC
antilinear-R	AGTCGACCTGCAGAAGAGGAT
Up-F	CGATGCTCAAGATGATCAATAC
Up-R	CATTGTTATATCGGCTATAAGGGTTAC
Down-F	CATCTAGTGTCATATTAACAGCAATG
Down-R	GAATGGAACAATTACACAATTACCG
NcoI-bsh1	catgCCATGGGGTGTACTGCCATAACTTATCAATCTTA
SacI-bsh1	cGAGCTCTTAGTTAACTGCATAGTATTGTGCTTCTG
pNZ8148F	TAATGTCACTAACCTGCCCCGT

Table S4. Dietary composition and energy density in animal experiments.

Product	D12450J (LFD)		D12492 (HFD)[a]	
	**gm%**	***kcal*%**	**gm%**	***kcal*%**
Protein	19.2	20.0	26	20.0
Carbohydrate	67.3	70.0	26	20.0
Fat	4.3	10.0	35	60.0
**Ingredient**	**gm**	***kcal***	**gm**	***kcal***
Casein, 30 Mesh	200	800	200	800
L-Cystine	3	12	3	12
Corn Starch	506.2	2024.8	0	0
Maltodextrin 10	125	500	125	500
Sucrose	68.8	275.2	68.8	275.2
Cellulose, BW 200	50	0	50	0
Soybean Oil	25	225	25	225
Lard	20	180	245	2205
Mineral Mix S10026	10	0	10	0
Dicalcium Phosphate	13	0	13	0
Calcium Carbonate	5.5	0	5.5	0
Potassium Citrate·$1H_2O$	16.5	0	16.5	0
Vitamin Mix V10001	10	40	10	40
Choline Bitartrate	2	0	2	0
FD&C Yellow Dye #5	0.04	0	0	0
FD&C Red Dye #40	0	0	0	0
FD&C Blue Day #1	0.01	0	0.05	0
**Total**	1055.05	4057	773.85	4057
**kcal/gm**	3.85		5.24	

[a] As for D12492, the content of cholesterol is 279.6 mg/kg.

Table S5. Mice average daily energy intake during the modeling period (1-12 weeks).

	Average energy intake （kcal·day^{-1} per mouse）	
**Weeks**	**LFD**	**HFD**
**1**	14.49	14.85
**2**	13.08	13.40
**3**	12.80	13.67
**4**	12.39	13.86
**5**	12.60	13.59
**6**	12.88	13.87
**7**	13.07	14.11
**8**	12.27	13.99
**9**	12.76	14.77
**10**	12.60	15.24
**11**	12.55	14.48
**12**	12.62	16.50

Table S6. Mice average daily energy intake during treatment (13-20 weeks).

Weeks	Average energy intake (kcal·day^{-1} per mouse)			
	**LFD**	**HFD**	**HFD+Lp20-WT**	**HFD+Lp20-*Δbsh1***
**13**	12.77	15.01	14.09	14.32
**14**	13.20	15.45	15.55	14.84
**15**	11.35	14.94	15.66	15.29
**16**	11.74	14.73	15.31	15.07
**17**	12.18	14.33	14.72	14.33
**18**	12.36	15.42	14.98	13.94
**19**	12.79	14.56	14.30	14.75
**20**	12.52	15.12	14.75	14.41

Table S7. Weight trajectories and Area Under the Curve (AUC) for individual mice during the modeling period (Weeks 1-12).

Weeks	0	1	2	3	4	5	6	7	8	9	10	11	12	AUC
LFD	27	28.17	28.91	29.9	30.44	30.63	30.34	30.64	30.9	31.16	31.85	32.64	33.39	365.775
	26.49	27.64	28.35	29.35	31.08	32.12	32.64	32.52	32.83	33.62	33.79	35.57	36.76	381.135
	28.07	28.82	29.52	30.4	30.98	31.79	32.06	32.6	32.71	32.66	33.81	33.72	34.21	380.21
	26.72	27.82	28.77	29.53	29.15	29.61	30	30.22	30.76	30.47	31.97	32.05	32.67	360.045
	27.09	27.8	28.74	28.77	29.03	29.28	29.95	30	29.64	29.32	29.62	31.05	31.22	352.355
	28.82	30.46	30.77	31.98	31.89	32.16	32.1	32.9	32.07	32.87	32.75	33.63	34.37	385.175
	27.83	28.77	30.87	32.05	33.47	35.43	37.16	37.45	38.35	40	40.75	42.5	44.74	433.085
	27.85	28.25	29.71	31.27	32.11	33.38	33.46	34.62	32.92	33.36	34.65	37.14	38.35	393.97
	27.04	28.78	29.57	31.83	32.24	33.51	34.27	35.05	35.35	36.88	37.57	39.83	41.59	409.195
	29.25	29.9	32.33	33.45	34.65	36.01	38	38.47	39.74	40.94	42.22	44.42	46.27	447.89
	27.23	29.61	30.29	31.74	31	31.11	32.4	33.26	34.42	34.88	35.9	37.08	39.36	394.985
	26.79	27.91	28.86	30.43	31.76	33.06	34.1	35.26	36.38	37.33	39.36	41.23	42.76	410.455
	27.64	29.4	30	32.07	33.15	33.93	34.82	35.34	36.4	37.03	38.28	39.82	42.03	415.075
HFD	28.14	29.52	30.73	31.23	31.39	31.51	32.35	33.53	34.24	35.31	35.6	36.04	37.34	394.19
	27.44	28.2	28.92	30.35	31.15	31.57	32.74	33.86	34.53	35.66	36.14	37.74	39.2	394.18
	27.59	28.35	28.36	30.24	30.95	32.26	32.84	33.51	33.75	35.19	36.61	38.16	39.68	393.855
	27.05	29.13	29.3	30.82	31.52	32.31	32.77	33.65	34.31	35.69	36.84	38.35	38.36	397.395
	28.4	30.22	31.13	32.98	33.6	34.16	33.56	34.2	35.49	36.52	36	37.63	39.14	409.26
	27.88	30.13	31.77	32.25	32.78	34.47	35.97	37.05	37.67	38.42	39.54	41.71	43.23	427.315
	27.73	28.64	30.46	31.58	32.21	33.51	33.89	34.12	34.88	36.42	37.91	39.54	41.67	407.86
	27.36	29.38	30.1	31.18	31.44	32.91	33.65	33.94	34.84	35.49	36.92	38.69	39.7	402.07
	27.66	29.89	30.91	31.55	32.06	32.76	33.86	35.33	36.41	36.15	37.83	40.08	40.79	411.055
	27.92	29.15	30.35	32.07	32.66	32.76	33.6	34.36	34.99	35.91	36.73	38.33	40.07	404.905
	28.02	29.72	30.15	31.42	32.12	33.76	34.4	36.62	36.82	38.46	39.88	42.26	44.29	421.765

Table S8. Weight trajectories and Area Under the Curve (AUC) for individual mice during treatment (13-20 weeks).

Weeks	13	14	15	16	17	18	19	20	21	22	AUC
LFD	33.39	32.81	32.47	32.08	32.52	32.1	32.39	31.3	31.78	32.44	290.365
	36.76	36.88	37.26	36.02	36.32	37.36	37.13	35.9	33.99	35.58	327.03
	34.21	33.88	34.25	33.48	33.68	33.77	34.37	34.29	34.05	34.82	306.285
	32.67	32.37	32.39	31.65	31.93	31.65	33.18	31.59	31.98	32.78	289.465
	31.22	30.91	31.17	31.03	31.55	30.81	31.64	31.5	32.69	32.32	283.07
	34.37	33.98	33.61	33.39	34.08	33.32	33.34	33.75	33.89	34.75	303.92
HFD	44.81	44.42	47.81	50	52.32	53.16	53.34	53.35	52.01	52.23	454.93
	41.88	43.4	44.99	45.91	47.75	48.98	50.41	50.38	49.88	49.23	427.255
	40.81	43.12	44.32	45.53	47.33	47.52	48.64	49.19	48.67	47.8	418.625
	47.46	48.88	50.05	51.75	53.37	53.8	54.44	55.94	55.6	55.29	475.205
	43.32	43.11	46.29	48.76	51.93	54.5	55.34	57.06	56.32	55.89	462.915
	39.31	40.07	40.9	41.81	43.3	45.25	46.82	49.26	48.57	48.64	399.955
Lp20	43	41.58	42.06	44.03	45.54	47.46	47.13	46.24	45.76	43.68	403.14
	40.14	41.7	42.5	43.29	44.45	46.4	47.44	47.25	46.69	46.92	403.25
	39.93	38.72	39.39	41.65	42.69	44.6	46.72	45.98	44.75	45.22	387.075
	42.68	38.2	38.13	39.75	42.71	44.75	47.69	49.48	47.94	47.23	393.605
	40	34.65	36.12	37.52	39.85	41.36	39.87	41.59	41.09	41.25	352.675
	38.83	35.07	36.6	38.12	39.13	39.1	40.26	40.97	40.13	40.34	348.965
Lp20*Δbsh*	38.89	37.78	36.8	38.57	41.71	42.4	45.69	48.38	46.25	47.58	380.815
	41.87	40.28	40.97	42.66	46.68	45.33	47.88	48.87	48.56	49.51	406.92
	39.78	40.85	42.08	44.05	46.38	48.32	50.62	51.2	50.84	50.34	419.4
	39.92	42.29	44.94	46.14	48.4	48.02	49.33	49.82	49.63	49.95	423.505
	40.62	42.13	44.79	47.17	49.63	49.94	50.68	51.14	50.48	50.82	431.68
	44.92	46.57	48.06	49.41	49.55	50.02	49.38	50.17	50.02	49.84	440.56

Table S9. NAS scoring of NASH.

Histological Features	Scoring	Diagnostic Criteria
**Steatosis**	0	<5%
	1	5%~33%
	2	34%~66%
	3	>66%
**Hepatocellular ballooning**	0	None
	1	Minimal
	2	Moderate with prominence
**Lobular inflammation (all inflammatory foci)**	0	None
	1	<2 foci
	2	2--4 foci
	3	>4 foci
**NAS total score**	0--8	

Table S10. Liver weight and liver index (Mean and standard deviation) of mice after 8 weeks of intervention.

Group	Liver weight(g)		Liver index (%)	
	Mean	SD	Mean	SD
LFD	1.19^{c}	0.10	3.93^{a}	0.20
HFD	1.97^{a}	0.23	4.01^{a}	0.35
Lp20	1.20^{c}	0.19	2.88^{b}	0.29
Δbsh1	1.68^{b}	0.14	3.56^{a}	0.24

a,b,c,d Different letters in the same column represent significant differences ($P < 0.05$).
